# Exploring Gender Moderation: The Impact of Neighborhood Factors on Adolescent Internalizing and Externalizing Symptoms

**DOI:** 10.3390/children11040389

**Published:** 2024-03-25

**Authors:** Fei Pei

**Affiliations:** School of Social Work, David B. Falk College of Sport and Human Dynamics, Syracuse University, White Hall, 440, Syracuse, NY 13244, USA; fpei01@syr.edu; Tel.: +1-(732)-429-6697

**Keywords:** neighborhood, adolescent behavior problems, gender differences, structural equation modeling

## Abstract

Limited previous studies investigated the influences of various types of neighborhood factors on adolescent behavior problems. Meanwhile, although previous theoretical frameworks suggested that gender played a significant role in terms of neighborhood impacts on adolescent behavioral problems, few studies investigated the gender differences in such neighborhood influences. Using the year 9 and year 15 data of the national dataset Future of Families and Child Wellbeing Study (FFCWS, overly sampled participants from low-income families), this study examined how the neighborhood structural and process factors can affect adolescent behavioral problems (internalizing and externalizing symptoms) and whether gender worked as a significant moderator for such relationships in the U.S. Structural equation models and multigroup SEM were estimated (N = 3411). Findings suggested that residential instability was associated with increased levels of internalizing symptoms among adolescents at age 15, whereas neighborhood social cohesion was linked to reduced levels of externalizing symptoms throughout adolescence. Furthermore, the moderating effects of gender were found for the association between residential instability and internalizing symptoms. Implications of such findings are further discussed.

## 1. Introduction

In recent decades, there has been a notable increase in research focusing on how neighborhood factors impact behavioral problems [1,2]. Neighborhood structural characteristics, referring to the economic and ethnic characteristics of an adolescent’s residential context [3], were found to be associated with adolescent internalizing and externalizing symptoms [4]. According to neighborhood disorganization theory [3], exposure to dangerous neighborhood structural characteristics can increase the risk of developing adolescent behavioral problems [5]. Moving beyond neighborhood structural characteristics, the influences of neighborhood process factors on adolescent internalizing and externalizing symptoms were also discussed in empirical studies [6]. Neighborhood process factors refer to the resources and relationships among residents. It reflects whether residents share the same values and get along with each other. However, limited studies have discussed how the two types of neighborhood factors work together to influence adolescent internalizing and externalizing symptoms.

Gender differences were found to be critical in the studies of neighborhood influences [7]. Because the conceptualization of gender is developed from social experiences, neighborhood experiences for boys and girls are often different [8]. Previous studies suggested that compared to boys, girls can adapt to changes in their lives more easily [9]. Meanwhile, boys are at higher risk of getting involved in criminal activities and misbehaviors because they want to show their masculinity and connect to their peers. Other research claimed that such masculinity is more likely to result in the mistreatment of young women in disadvantaged communities [10,11]. Building on this opinion, the investigation of gender differences in the influence of these two different neighborhood factors should be emphasized. Therefore, this study sought to examine how gender moderates the influences of two types of neighborhood factors (neighborhood process factor and structural factor) on adolescent internalizing and externalizing symptoms in the U.S.

### 1.1. Neighborhood Factors and Adolescent Behavioral Problems

Because adolescence involves complicated developmental processes such as rapid physical development, brain development, and puberty, problematic behaviors (e.g., aggression, failure in school, and delinquency) in adolescence were commonly investigated in previous studies [12]. The behavioral patterns developed in adolescence can last into adulthood and have long-term influences [13]. According to the World Health Organization [14], 10% to 20% of adolescents between ages 10 and 19 experience mental health issues and behavioral disorders, which is the second leading cause of disease among young adolescents aged 10 to 14. Adolescent behavioral problems can be further categorized as internalizing and externalizing symptoms [15,16]. Internalizing symptoms include inward-directed symptoms such as depression, withdrawal, and anxiety, whereas externalizing symptoms are more related to aggressive behaviors or verbal abuse toward others such as disruptive behaviors, breaking the rules, and interpersonal-violent behaviors [17].

Various predictors have been suggested to affect adolescent behavioral problems including individual experiences (e.g., child maltreatment), family factors (e.g., parent–child attachment, domestic violence), and environmental and school factors (e.g., neighborhood, peer relationships) [18,19,20]. Compared to younger children, adolescents still depend on their parents but also start to move toward independence. For example, a study found that approximately half of teenagers aged 15 to 19 spent over 90% of their daily time within their neighborhoods every day [21]. Thus, neighborhood spaces may affect adolescents more compared to younger children.

An increasing number of empirical and theoretical studies suggest a strong relationship between neighborhood factors and problematic behaviors in adolescents [22,23,24,25]. Various types of neighborhood factors were discussed in previous studies, with some discussing the environmental characteristics of a community (e.g., percentage of residents living in poverty) and others focusing on the interactive relationships among neighbors (e.g., whether you can get alone with your neighbors.) For example, among 571 urban African American adolescents aged around 17, higher neighborhood poverty rates and residential instability was associated with greater adolescent internalizing symptoms [26]. Some qualitative studies in Europe discussed community gang problems and adolescent safety [27], as well as the contextual framework for child abuse prevention [28]. Furthermore, various types of neighborhood factors influence individuals’ behaviors through different mechanisms. Social disorganization theory is a pioneering theory that was applied to analyze the influences of neighborhood structural factors on high-risk behaviors in the early 20th century [3]. This theory posits that growing up in neighborhoods with disadvantaged economic sources, high ethnic heterogeneity (more ethnic diversity), and high residential instability leads to the disruption of neighborhoods and further creates conditions favorable to crime and adolescent behavioral problems [29,30]. In the past decade, criticism has emerged regarding the ethnic heterogeneity aspect of this theory, particularly in light of the changing racial composition of the U.S. [31]. Even though some empirical studies found that neighborhood structural factors did not have direct effects on adolescent internalizing or externalizing symptoms [32,33,34], a majority body of empirical studies suggested neighborhood structural factors can affect adolescent behavior problems [35,36]. A study with 2805 Chicago children between 5 and 11 years old suggested that concentrated disadvantage, including poverty rate, residential stability, and ethnic heterogeneity, were positively associated with adolescent internalizing behavioral problems (depression, anxiety, withdrawal, and somatic problems) after accounting for family-level factors [37]. Similarly, Sundquist et al. [38]. found that neighborhood contexts including low education level, low family income, unemployment status, and receipt of social welfare were associated with increased risks of externalizing and internalizing disorders.

Sampson and colleagues extended this theory to emphasize the role of social interactions in the neighborhood context (neighborhood process factors) [6,39,40]. Collective efficacy theory, one of the most commonly discussed theories regarding neighborhood process factors, emphasizes the functions of informed social control and social cohesion on residents’ behaviors. The theory suggests that neighborhoods with less social capital have a harder time maintaining common values and social controls to foster safety [39,41].

Empirical research yielded varied results regarding the impact of neighborhood process factors on adolescent behavioral problems. Some prior studies suggested neighborhood process factors can benefit adolescent development [42,43]. A cross-sectional study involving 1538 African American youths aged 10 to 18 residing in inner-city neighborhoods indicated that neighborhood social capital inversely and significantly affected adolescent depression [30]. Similarly, utilizing a nationally representative sample of 5183 Canadian adolescents, Kingsbury et al. [42] suggested that increased neighborhood social cohesion can buffer the impact of life stressors on both internalizing and externalizing symptoms among adolescents. However, other studies indicated that neighborhood process factors were not associated with adolescent internalizing and externalizing symptoms [1]. A study of 2232 children aged 5 to 10 claimed that neighborhood collective efficacy did not significantly predict changes in antisocial behavioral problems [44]. More empirical studies are needed to clarify the relationships between neighborhood process factors and adolescent behavioral problems. Furthermore, studies that systematically examine the influence of both neighborhood structural and process factors on child behavioral problems are necessary.

### 1.2. Neighborhood Influences and Gender Differences

Neighborhood factors may have varying effects on behavioral problems among boys and girls due to the differences in their exposure to neighborhood environments. To understand the mechanisms behind neighborhood effects on adolescent internalizing and externalizing symptoms, this study investigated gender as a moderator within these relationships. According to the American Psychological Association [45], gender refers to the “socially structured roles, behaviors, and activities that a given society considers appropriate for boys and men or girls and women”. As this study discussed the gender differences during early adolescence, in which individuals are in their early stage of development of gender identification, the current study follows the boys’ and girls’ definitions in APA. Further examination of nonbinary gender differences is needed. 

Gender is one of the strongest and most consistent factors associated with internalizing and externalizing symptoms, but findings have been inconsistent [46,47,48]. Rocchino et al.’s study [47] found that gender predicted adolescent internalizing symptoms but not externalizing symptoms, whereas Gauthier-Duchesne et al. [49] suggested that girls showed a higher likelihood of experiencing increased internalizing symptoms, whereas boys demonstrated a higher likelihood of experiencing increased externalizing symptoms.

Although gender differences have been discussed in previous research on internalizing and externalizing symptoms, they have been under-examined in the traditional neighborhood literature. Traditional social disorganization theory did not emphasize gender differences. Some scholars emphasized the unique neighborhood experiences for different genders [8,50,51]. Because the expectations of socialization for boys and girls are different in society, parents tend to closely monitor and restrict girls’ activities and give more freedom to boys [52]. Few studies have explored gender differences in neighborhood influences, and findings have indicated mixed effects. For neighborhood structural factors, some studies found that compared with boys who spent more time outside, girls between 14 and 16 years old were less likely to be exposed to neighborhood violence [8]. Thus, boys showed more severe behavioral problems [53]. However, some empirical studies found no gender differences in terms of exposure to disadvantaged neighborhoods [54], and neighborhood disadvantage was found to be associated with increased violent behaviors among both boys and girls [55,56].

As for neighborhood process factors, some scholars argued that because girls are more sensitive to social support and fearful of criminal events, such factors may show stronger influences on girls than boys [57]. Similarly, some studies suggested that girls living in neighborhoods with low collective efficacy showed greater problematic behaviors [58]. However, other studies suggested that girls are less sensitive to their living environment than boys due to less exposure to the environment [53], which is supported by Kim’s empirical study of 589 adolescents aged 12 to 17 [59]. Considering the insufficiency of and inconsistencies in the literature, research regarding the moderating effects of gender is needed.

### 1.3. Present Study

**Figure 1:**
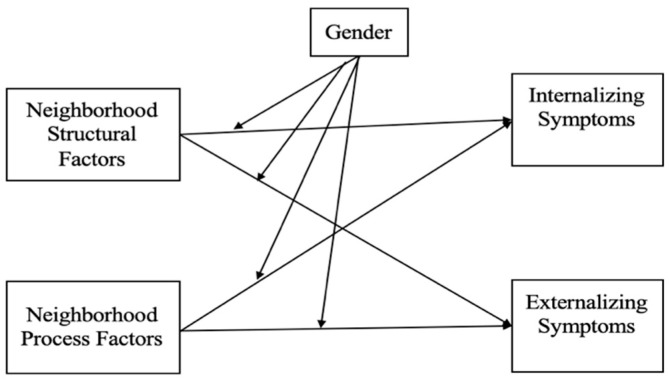
Conceptual model.

Building on previous literature, this study examined the influences of two types of neighborhood factors (neighborhood structural and process factors) on adolescent behavioral problems and the moderating effects of gender in these relationships. The conceptual model of this specific empirical study is displayed in Figure 1. The following research questions were addressed: (1) Are the two types of neighborhood factors associated with adolescent internalizing and externalizing symptoms? (2) Do these associations differ for boys and girls? I hypothesized that (a) living in disadvantaged neighborhoods and those with low collective efficacy would predict more severe adolescent internalizing and externalizing symptoms; and (b) boys would be more sensitive to neighborhood structural factors, whereas neighborhood process factors would have the same effects on boys and girls.

## 2. Method

### 2.1. Data and Sample

The present study used the U.S. data from the age 9 and age 15 restricted files of the national dataset Future of Families and Child Wellbeing Study (FFCWS). FFCWS is a longitudinal study following 4898 children from birth to age 15 that purposely over-sampled families in poverty or at risk [60]. This is a national dataset that was collected in the U.S., and a stratified and multistage sampling strategy was applied [61]. Children were randomly selected from hospitals located in 20 large cities in the U.S. at their birth. Due to participant attrition, the Year 9 follow-up survey included 3630 children and was collected in 2007–2010 (children aged 9 in 2007). Year 15 follow-up survey included 3580 children and was collected between 2014 and 2017 (children aged 15 in 2014) [62].

Using the Year 9 and Year 15 datasets, I examined the relationship between neighborhood structural and process factors (child aged 9) and adolescent behavioral problems (child aged 15) and the moderating effects of gender. The current study featured 3411 children as 169 children did not furnish data for any of the key variables considered in this study.

### 2.2. Measures

#### Neighborhood Structural Factors

Theoretically, neighborhood structural factors can be categorized using three main indexes: economic disadvantage, residential instability, and ethnic heterogeneity [3]. Nine items about neighborhood structural factors were collected at the focal child’s age of 9 (census track level). To deal with multicollinearity (as shown in Table 1), principal component analysis (PCA) was utilized to develop the three indexes [63,64]. An economic disadvantage was constructed using five items: the proportion of non-Hispanic Black residents (*M* = 35%, *SD* = 35.70), the proportion of families below the federal poverty line (*M* = 15%, *SD* = 13.10), the proportion of civilian labor force (aged 16 or older) that is unemployed (*M* = 9%, *SD* = 6.90), the proportion of the population with an education level less than a bachelor’s degree (*M* = 81%, *SD* = 15.20), and the proportion of households receiving public assistance (*M* = 6%, *SD* = 6.30) [1,40]. Specifically, based on the findings from PCA, the proportion of non-Hispanic Black residents serves as an indicator of economic disadvantage, aligning with previous research that links the proportion of Black residents with the economic condition of a neighborhood [65].

Residential instability was measured using the proportion of renter-occupied homes (*M* = 44%, *SD* = 24.30). Ethnic heterogeneity was indexed using the proportion of Latinos (*M* = 18%, *SD* = 24.10), Asians (*M* = 4%, *SD* = 7.90), and foreign-born residents (*M* = 12%, *SD* = 13.70). 

### 2.3. Neighborhood Process Factors

Two latent neighborhood process factors were assessed when the child was 9 years old using two scales: the Informal Social Control Scale (e.g., residents are willing to help their neighbors; people in the neighborhood do not share the same values) and the Social Cohesion and Trust Scale (e.g., neighbors get involved if buildings were spray painted) [41]. Primary caregivers rated these eight items on a 4-point scale from 1 (*strongly agree* or *very likely*) to 5 (*strongly disagree* or *very unlikely*). To ensure higher scores reflected stronger social cohesion and social control within the neighborhood, seven items were reverse-coded. Social cohesion captured the shared values and strong interpersonal bonds among residents, while social control represented the community’s willingness to intervene for the common good. Cronbach’s alpha for the social cohesion and social control scales in this sample were 0.88 and 0.80, respectively.

### 2.4. Adolescent Behavioral Problems

Adolescent behavioral problems were measured using two subscales of the Child Behavior Checklist (internalizing and externalizing symptoms) among 15-year-olds [66,67]. Internalizing symptoms were assessed using the internalizing subscale, including anxious or depressed and socially withdrawn behaviors, and externalizing symptoms were measured using the externalizing subscale, including aggressive and rule-breaking behaviors. Caregivers were interviewed to rate their children’s behaviors from 1 (*not true*) to 3 (*often true*). By adding scores for each item, an internalizing sum score and an externalizing sum score were calculated, with higher scores suggesting more severe internalizing and externalizing symptoms (internalizing scale α = 0.79; externalizing scale α = 0.85).

### 2.5. Gender

The gender of participants was measured at birth (0 = *male*, 1 = *female*).

### 2.6. Control Variables

Demographic variables—including the child’s race, household size, family income, mother’s education level, and marital status—were added as control variables. The education level and marital status of the participants’ mothers were categorical variables and dummy-coded. Child maltreatment experiences were controlled in this study and measured using the Parent–Child Conflict Tactics Scales for children aged 9 [68]. Each maltreatment type (i.e., emotional, physical, and neglect) had a sum score, with higher scores suggesting more severe maltreatment. Peer bullying was added as a control variable and measured at youth aged 15 using a 5-item scale. A sum score was calculated, with higher scores indicating worse peer bullying. Child behavioral problems at age 9 were added as a control variable because a history of child behavioral problems can be a risk factor for later behavioral problems.

### 2.7. Analytic Strategy

First, a measurement model was examined using Mplus 8.0 [69]. Then, structural equation modeling (SEM) using full maximum likelihood was conducted to examine the correlation between two types of neighborhood factors (neighborhood structural and process factors) and adolescent internalizing and externalizing symptoms. Additionally, the moderating effects of gender on the relationships between two neighborhood factors and adolescent behavioral problems were examined using the multigroup SEM model. The sample was divided into two subgroups according to gender. The research models for boys and girls were estimated separately, and the standardized pathways for each subgroup were calculated in each model. Model fit indexes were the comparative fit index (CFI), the root mean square error of approximation (RMSEA), and the standardized root mean square residual (SRMR). A CFI above 0.95, a RMSEA less than or equal to 0.05 (confidence interval [CI] lower value near 0 and upper value ≤0.05), and a SRMR below 0.05 indicate an acceptable model fit [70,71].

## 3. Results

Table 2 provides a summary of the descriptive statistics for the observed variables. This study featured a sample of 3411 adolescents; 47.82% (1631) were girls and approximately half were Black, followed by Hispanic (27.30%), White (21.10%), and other (4.00%). Approximately 25.13% of these adolescents’ primary caregivers’ educational achievement was less than high school, and only 25.15% of primary caregivers held a college degree. The average household income-to-poverty ratio was 199% (lower than 100% indicated living in poverty), and 29.52% of adolescents’ mothers were married to the adolescents’ biological fathers at adolescents aged 9. On average, each family had approximately two children in the household. The mean scores of the focal adolescents’ types of child maltreatment experiences were 7.13 (*SD* = 5.25) with a theoretical range of 0–30 for psychological aggression, 2.48 (*SD* = 4.17) with a range of 0–27 for physical assault, and 0.71 (*SD* = 1.91) with a range of 0–25 for neglect, with higher values indicating more severe child maltreatment experiences. The mean sum score of the focal adolescents’ internalizing symptoms at age 9 was 37.03 (*SD* = 5.70) with a range of 32–96, and the mean sum score of externalizing symptoms at age 9 was 41.22 (*SD* = 6.92) with a range of 35–105. Higher scores suggested more severe behavioral problems. At the focal adolescents’ age 15, the average sum score of internalizing and externalizing symptoms were 10.04 (*SD* = 2.45) with a range of 8–23 and 24.39 (*SD* = 5.06) with a range of 20–56, respectively.

### 3.1. SEM Model

Due to the two latent variables (neighborhood social cohesion and social control) involved in this study, an SEM was estimated using Mplus 8.0 (Table 3) [69]. The model fit indexes were acceptable: RMSEA = 0.03 (90% CI = 0.030, 0.034), CFI = 0.95, and SRMR = 0.055. As shown in Table 3, gender and social cohesion (latent variable) were critical predictors of both adolescent internalizing and externalizing symptoms (Model 1). Compared to boys, girls were at higher risk of experiencing internalizing symptoms (β = 0.07, *p* < 0.001) but lower risk of experiencing externalizing symptoms (β = −0.04, *p* = 0.02). Higher neighborhood social cohesion was significantly associated with both lower internalizing (β = −0.06, *p* = 0.048) and externalizing (β = −0.07, *p* = 0.02) symptoms, after controlling for other covariates. However, no notable correlations were observed between neighborhood structural factors, social control, and adolescent internalizing and externalizing symptoms.

### 3.2. Moderation Model

To examine gender differences in the impacts of the two types of neighborhood factors (neighborhood structural and process factors), a multigroup SEM was estimated, and pathways for boys (*n* = 1780) and girls (*n* = 1631) were estimated separately in Model 2 (Table 3). The model fit was acceptable, with RMSEA = 0.03, CFI = 0.95, and SRMR = 0.06. Results suggested that there are gender differences in how neighborhood factors influence adolescent behavioral problems. The influences of neighborhood social cohesion were significantly different for boys and girls. Higher neighborhood social cohesion was significantly associated with reduced levels of both internalizing and externalizing symptoms in boys, but not in girls (boys’ internalizing symptoms: β = −0.10, *p* = 0.02; boys’ externalizing symptoms: β = −0.10, *p* = 0.02; girls’ internalizing symptoms: β = −0.02, *p* = 0.60; girls’ externalizing symptoms: β = −0.04, *p* = 0.43). Moreover, living in neighborhoods characterized by increased levels of residential instability was associated with higher levels of internalizing symptoms solely among girls, with no significant effect observed among boys (girls’ internalizing symptoms: β = 0.08, *p* = 0.005; boys’ internalizing symptoms: β = 0.04, *p* = 0.10).

## 4. Discussion

This study investigated the correlation between various neighborhood factors and adolescent internalizing and externalizing symptoms, as well as any gender differences within this relationship. Analyzing data from at-risk families in the U.S., this study found different types of neighborhood factors were associated with different adolescent internalizing and externalizing symptoms, and gender played a moderating role in the influences of residential instability and social cohesion, thereby enriching neighborhood research by highlighting gender-specific differences.

In line with previous empirical research [49,72], findings in this study suggest that compared to boys, girls were less likely to experience externalizing symptoms. According to research in endocrinology, compared to girls, boys in puberty are motivated by rapidly increased testosterone and tend to impulsively act out with physical aggressions [73,74]. Similar results were found in Whittle et al.’s research [75], in which neighborhood disadvantage was associated with brain development from early to late adolescence. Moreover, girls were more likely to experience internalizing symptoms than boys, which is consistent with evidence from prior empirical studies wherein girls were found to be more vulnerable to internalizing symptoms than boys [49]. According to Mendle et al.’s study [76], girls have higher emotional reactivity during puberty, which increases their likelihood of experiencing internalizing symptoms.

Interestingly, after adding gender as a moderator, neighborhood social cohesion was only associated with boys’ adolescent behavioral problems, which contradicts previous research in which neighborhood process factors showed stronger influences on girls’ behavioral problems [57]. Compared to girls, boys may spend more time in the neighborhoods they live in once they reach adolescence, which makes them more vulnerable to their living environment. More positive role models exist in neighborhoods with high social cohesion, and residents in such neighborhoods are more willing to help each other, which consequently decreases boys’ conduct problems [53]. Moreover, social control measured for the children aged 9 did not have significant effects on either adolescent internalizing or externalizing symptoms at age 15. These findings support the perspective that social control may have a strong immediate effect but cannot last long enough to affect adolescent behavioral problems at age 15 [77,78]. However, further research is needed to provide a deeper understanding of social control.

A moderating effect between gender and neighborhood residential instability was found for girls’ adolescent internalizing symptoms. Compared to boys, girls were more sensitive to residential instability, which means that living in neighborhoods with high residential instability increased the possibility that girls would experience internalizing symptoms more than that for boys. Such a finding conflicts with certain previous research, which concluded that a high level of residential instability was not a significant predictor of either internalizing or externalizing symptoms [32,34]. A potential explanation may be that living in neighborhoods characterized by high levels of residential instability brings frequent changes to adolescents’ living environment, and the need to frequently adjust to a new environment might lead to a higher likelihood of mental health issues among girls, who are more sensitive to their living environment and have higher levels of emotional reactivity during puberty [76].

### 4.1. Strengths and Limitations

There are several limitations in this study. First, because the FFCWS dataset included many children who were born in single-parent families, the generalizability of the findings from this study is limited. Also, most of the key variables in this study were reported by caregivers, which may cause mono-information bias. The 15-year-old adolescents might have different perspectives of their neighborhood process factors (neighborhood structural factors are national objective factors), which might lead to bias. Also, due to the relatively small intraclass correlation value (ICC < 0.2), the current study is not able to capture the cluster effects of neighborhood factors. Further studies are needed to fulfill this gap. However, this study has some notable strengths. The FFCSW dataset is a relatively new dataset; Year 15 data were collected in 2018. This study reflects the most up-to-date information on neighborhood influences. Second, this is the first study to systematically examine the interactional effects between gender and different types of neighborhood influences on adolescent behavioral problems, which expands the existing literature on how community factors can affect child development. Finally, a relatively large sample size used in this study minimized sampling errors and built a rigorous estimation of the moderation model.

### 4.2. Implications and Conclusions

The findings of this study enhance the existing literature by emphasizing the gender differences in various neighborhood factors, as well as their interactive effects on adolescent behavioral issues. These findings offer valuable insights for practitioners operating at both micro and macro levels. Adolescence is a complex and dynamic developmental stage in which various types of problems related to gender and neighborhood factors can occur. Regarding clinical services for children with internalizing or externalizing disorders, practitioners should pay extra attention to clients’ gender differences, especially adolescents with externalizing symptoms [79]. The diagnosis of externalizing symptoms in boys without considering the living environment and context should raise practitioners’ attention and signal the need for further investigation of the individual’s community/neighborhood environment [80]. Furthermore, neighborhood social cohesion, a critical correlation of adolescent internalizing and externalizing symptoms, should be emphasized in community-based intervention and prevention efforts for boys. Prevention programs that build a supportive and closely connected neighborhood environment can significantly improve the social cohesion of communities, which can directly decrease internalizing and externalizing symptoms among boys living in these communities. Additionally, since the changes in neighborhood structural factors often require significant effort and are challenging to achieve, enhancing the development of protective factors may offer a more effective approach. For example, interventions that promote the bonds among neighbors, and community activities like barbeques and summer pool nights might significantly change the neighborhood process factors. When practitioners deliver such community-level interventions, they should carefully examine clients’ living environments to accurately and effectively apply these community-level interventions. In particular, for communities with high residential instability, clinical services or neighborhood interventions specifically designed for girls can effectively decrease internalizing symptoms. For example, informational programs that introduce neighborhood environments and resources to new teenage girl residents can benefit the healthy development of these new residents.

## Figures and Tables

**Table 1:** Correlation matrix for observed neighborhood Items.

	1	2	3	4	5	6	7	8
1. Non-Hispanic Black population								
2. Families below federal poverty line	0.52 *							
3. Unemployed population	0.58 *	0.80 *						
4. Population with less than a bachelor’s degree	0.40 *	0.59 *	0.54 *					
5. Households on public assistance	0.49 *	0.85 *	0.78 *	0.55 *				
6. Renter-occupied homes	0.33 *	0.64 *	0.52 *	0.30 *	0.55 *			
7. Latinos	−0.39 *	0.21 *	0.11 *	0.24 *	0.20 *	0.20 *		
8. Asians	−0.26 *	−0.15 *	−0.16 *	−0.24 *	−0.06 *	0.04 *	0.11 *	
9. Foreign born	−0.30 *	0.02	0.11	−0.03	0.19 *	0.31 *	0.55 *	0.68 *

*Note.* * *p* < 0.01.

**Table 2:** Descriptive statistics of observed variables (*n* = 3411).

	*M* (*SD*)	%	Range
Gender (female)		47.82	
Race			
White		21.10	
Black		47.60	
Hispanic		27.30	
Other		4.00	
Primary caregiver’s education			
Less than high school		25.13	
High school or above		49.72	
College		25.15	
Household poverty level (%)	1.99 (2.28)		0–41
Mother married to child’s biological father		29.52	
Number of children in household	2.68 (1.33)		
Child’s maltreatment at age 9			
Psychological aggression	7.13 (5.25)		0–30
Physical assault	2.48 (4.17)		0–27
Neglect	0.71 (1.91)		0–25
Economic disadvantage (census tract, %)			
Non-Hispanic Black population	35 (35.70)		
Families below federal poverty line	15 (13.10)		
Unemployed population (aged 16 or older)	9 (6.90)		
Population without bachelor’s degree	81 (15.20)		
Households on public assistance	6 (6.30)		
Ethnic heterogeneity (census tract, %)			
Latino	18 (24.10)		
Asian	4 (7.90)		
Foreign born	12 (13.70)		
Residential instability (census tract)	44 (24.30)		
Child’s behavioral problems at age 9			
Internalizing symptoms	37.03 (5.70)		32–96
Externalizing symptoms	41.22 (6.92)		35–105
Child’s behavioral problems at age 15			
Internalizing symptoms	10.04 (2.45)		8–23
Externalizing symptoms	24.39 (5.06)		20–56

**Table 3:** Predictors of adolescent internalizing and externalizing symptoms.

	Model 1				Model 2 (Multigroup SEM)							
	Internalizing		Externalizing		Internalizing (Boys)		Externalizing (Boys)		Internalizing (Girls)		Externalizing (Girls)	
	β	95% CI	β	95% CI	β	95% CI	β	95% CI	β	95% CI	β	95% CI
Gender (female)	0.07 ***	0.03, 0.10	−0.04 *	−0.07, −0.01								
Neighborhood structural factors												
Economic disadvantage	−0.03	−0.07, 0.01	0.01	−0.03, 0.05	−0.03	−0.08, 0.03	−0.02	−0.07, 0.04	−0.03	−0.08, 0.02	0.05	−0.07, 0.11
Residential instability	0.02	−0.01, 0.07	−0.01	−0.04, 0.05	0.04	−0.02, 0.10	−0.04	−0.10, 0.02	0.08 **	0.02, 0.13	0.04	−0.02, 0.09
Ethnic heterogeneity	0.03	−0.01, 0.07	−0.03	−0.07, 0.02	−0.04	−0.09, 0.01	−0.04	−0.09, 0.01	0.02	−0.04, 0.08	−0.01	−0.08, 0.05
Neighborhood process factors												
Social cohesion	−0.06 *	−0.12, 0.00	−0.07 *	−0.13, 0.02	−0.10 *	−0.18, −0.02	−0.10 *	−0.18, −0.02	−0.02	−0.11, 0.06	−0.04	−0.13, 0.04
Social control	−0.01	−0.07, 0.04	0.04	−0.01, 0.09	−0.002	−0.08, 0.07	0.06	−0.02, 0.13	−0.03	−0.11, 0.05	0.02	−0.11, 0.05
*Control variables*												
Race												
Black	−0.21 ***	−0.26, −0.16	−0.03	−0.08, 0.01	−0.13 ***	−0.20, 0.06	−0.01	−0.07, 0.06	−0.28 ***	−0.35, −0.23	−0.07 *	−0.13, 0.00
Hispanic	−0.14 ***	−0.19, −0.09	−0.06 **	−0.10, −0.02	−0.11 **	−0.17, −0.04	−0.03	−0.09, 0.03	−0.17 ***	−0.24, −0.10	−0.10 **	−0.16, −0.04
Number of children	−0.03	−0.06, 0.01	0.04	0.00, 0.07	−0.03	−0.07, 0.02	0.04	−0.02, 0.09	−0.04	−0.08, 0.02	0.04	−0.01, 0.09
Household poverty level	−0.03	−0.08, 0.02	−0.03	−0.07, 0.00	−0.04	−0.12, 0.03	−0.04	−0.09, 0.00	0.00	−0.06, 0.05	−0.02	−0.06, 0.03
Marital status (married)	−0.06 **	−0.10, −0.03	−0.08 ***	−0.11, −0.05	−0.07 **	−0.12, −0.03	−0.10 **	−0.15, −0.06	−0.05	−0.10, 0.00	−0.07 **	−0.11, −0.02
Primary caregiver’s education												
High school or above	−0.02	−0.05, 0.01	−0.03 *	−0.07, 0.00	−0.01	−0.05, 0.04	−0.05 *	−0.09, 0.00	−0.04	−0.09, 0.01	−0.02	−0.07, 0.03
College	−0.01	−0.05, 0.01	−0.05 **	−0.08, −0.02	−0.01	−0.04, 0.07	−0.04	−0.08, 0.00	−0.04	−0.09, 0.02	−0.07 **	−0.11, −0.03
Peer bullying	0.14 ***	0.10, 0.19	0.11 ***	0.06, 0.15	0.28 ***	0.07, 0.19	0.08 **	0.02, 0.13	0.14 ***	0.07, 0.20	0.11 **	0.05, 0.17
Previous behavioral problems (age 9)	0.27 ***	0.21, 0.32	0.28 ***	0.23, 0.34	0.29 ***	0.20, 0.37	0.28 ***	0.20, 0.35	0.26 ***	0.18, 0.34	0.30 ***	0.22, 0.38
Child maltreatment	0.03	−0.02, 0.08	0.14 ***	0.08, 0.19	0.04	−0.03, 0.10	0.15 ***	0.08, 0.22	0.03	−0.03, 0.09	0.11 **	0.05, 0.17

* *p* < 0.05. ** *p* < 0.01. *** *p* < 0.001.

## Data Availability

Restrictions apply to the availability of these data. Data were obtained from the FFCWS research team and are available at https://ffcws.princeton.edu/data-and-documentation/restricted-use-contract-data/how-apply, accessed on 1 August 2019, with the permission of the FFCWS research team.
